# Enhanced Eugenol Composition in Clove Essential Oil by Deep Eutectic Solvent-Based Ultrasonic Extraction and Microwave-Assisted Hydrodistillation

**DOI:** 10.3390/molecules30030504

**Published:** 2025-01-23

**Authors:** Panawan Suttiarporn, Thananan Taithaisong, Samran Namkhot, Suwaporn Luangkamin

**Affiliations:** 1 Faculty of Science, Energy and Environment, King Mongkut’s University of Technology North Bangkok, Rayong Campus, Rayong 21120, Thailand; panawan.s@sciee.kmutnb.ac.th; 2 Department of Fundamental Science and Physical Education, Faculty of Science at Sriracha, Kasetsart University, Sriracha Campus, Chonburi 20230, Thailand; thanana2543th@gmail.com (T.T.); samran.n@ku.th (S.N.)

**Keywords:** deep eutectic solvent, essential oil, green extraction, microwave, eugenol, antioxidant activity

## Abstract

Eugenol is the key bioactive compound in clove oil, which has a variety of biological functions and is extensively employed in the medicinal and food industries. Nowadays, deep eutectic solvents (DESs) have received considerable attention as green solvents that enhance extraction efficiency. The present study investigated the effects of DESs on the eugenol composition in clove essential oils (CEOs) extracted from clove buds using ultrasonic- and microwave-assisted hydrodistillation techniques. The study revealed that both DES-based microwave-assisted hydrodistillation (DES-MHD) and ultrasonic-assisted DES pretreatment followed by microwave-assisted hydrodistillation (U-DES-MHD) significantly enhanced the eugenol purity in CEOs compared to the MHD method without the use of DESs. The great CEOs with a high amount of eugenol obtained via choline chloride–oxalic acid (ChCl-OA) at a 1:2 molar ratio were used as DESs. Their oils had a eugenol content of 82.90% and 83.34%, respectively, corresponding to the extraction by DES-MHD and U-DES-MHD methods, which were raised from the oil’s extraction without DES by MHD 7.42% and 8.36%, respectively. Corresponding to a strong antioxidant agent of eugenol, the oils extracted by ChCl-OA-based MHD and ultrasonic-assisted ChCl-OA-based MHD methods had significantly stronger DPPH radical scavenging activity with an IC_50_ level of 2.16 ± 0.11 and 2.19 ± 0.05 μg/mL, respectively, than the oils extracted without DESs. Hence, these innovative processes offer a promising approach to improving the bioactivity of clove oils, while providing straightforward operation and environmentally friendly extraction methods. Additionally, these novel processes may find application in other edible essential oil extractions for the food and pharmaceutical industries.

## 1. Introduction

The essential oil from clove buds is well-known and used for many medicinal purposes, including treatment of burns, contused wounds, pain relievers, tooth infections, and toothache. The biological activities of the oil provide health benefits, such as anti-inflammatory, analgesic, anesthetic, antinociceptive, and anticancer activity. The food industry uses clove essential oil as a food flavor and preservative, particularly in meat, due to its antioxidant and antimicrobial properties. It also serves as an ingredient in household products, cosmetics, and perfumes. The main bioactive ingredient in clove essential oil is eugenol, which contributes to the oil’s diverse biological activities [1,2,3]. Furthermore, the Food and Agriculture Organization (FDA) and World Health Organization (WHO) classify clove essential oil or eugenol as generally recognized as safe (GRAS) if they do not exceed the highest permissible daily intake for humans (2.5 mg/kg of body weight) [4]. Eugenol is a pale-yellow oil with a spicy aroma. A phenolic group in it showed strong antioxidant, antibacterial, antiviral, antifungal, anticancer, anti-diabetic, anti-inflammatory, and analgesic activities [4,5]. The quantity of eugenol in clove oil varies based on the genetic origin of the clove tree, growing regions, plant parts, and extraction methods [2,3,6].

Improving extraction technologies is an effective way to increase the yield and eugenol content in clove essential oil (CEO). Nowadays, some researchers are employing environmentally friendly techniques to extract bioactive substances from plants, including clove oil. Among them are microwave-assisted extraction (MAE), microwave-assisted hydrodistillation (MHD), ultrasonic-assisted extraction (UAE), a combination of ultrasound and microwave-assisted extraction (U-MHD) [6,7], and also, deep eutectic solvent (DES) extraction [8,9,10,11]. Their method offers a shorter extraction time, while improving both the quality and yield of the products. In contrast, conventional extraction techniques, such as hydrodistillation (HD), steam distillation (SD), and organic solvent extraction involve prolonged extraction times, elevated temperatures, significant waste from hazardous solvents, low efficiency, the thermal degradation of heat-sensitive components, and the potential contamination of extracts with toxic solvent residues [2,6,12,13].

Over the past decade, deep eutectic solvents (DESs) have become increasingly important in plant extraction processes, owing to their significant potential in producing plant extracts suitable for direct human consumption. In comparison to organic solvents, DESs are regarded as green solvents due to their low toxicity, biocompatibility, low volatility, non-flammability, thermal stability, as well as being inexpensive [14,15,16]. DESs are considered a novel kind of ionic liquid solvents, which are a two-component or three-component eutectic mixture. Deep eutectic solvents (DESs) encompass a variety of categories based on the nature of the complex agents [15]. The type of DESs that consist of quaternary ammonium salts as hydrogen bond acceptors (HBAs) and various hydrogen bond donors (HBDs), such as polyol, carboxylic acids, or amides, are mostly utilized for extracting essential oils and phenolic compounds from the plants [14,16]. When the HBAs and HBDs are mixed in certain molar ratios, the resulting mixture has a lower melting point than either of the individual parts. It can even be liquid at room temperature because of hydrogen bonding between the parts. The DESs, with their wide range of polarities and high solubility, have great potential as solvents for extracting natural compounds, particularly those that have a high hydrogen bond interaction with DES, such as phenolic compounds, which accelerates their release from plant tissue [16]. For this reason, DESs are widely used for extracting antioxidant compounds, including phenolic compounds, flavonoids, and anthocyanins, from plant materials.

In the past, researchers have utilized microwave-assisted DESs as a pretreatment extraction method and combined it with microwave-assisted hydrodistillation (DES-MHD) to extract essential oil from various plants. These include black and white pepper fruits [17], cumin seed [18,19], *Litsea cubeba* fruits [20], turmeric [21], and clove bud [8]. Additionally, researchers have applied ultrasonic-assisted DESs along with hydrodistillation (U-DES-HD) methods to extract essential oil from perilla leaves [22], as well as ultrasonic-assisted DESs along with microwave-assisted hydrodistillation (U-DES-MHD) methods to extract essential oil from cumin seeds [19] and *Schisandra chinensis* [23]. No researcher has reported using U-DES-MHD for the extraction of CEOs. Only DES-based ultrasonic-assisted extraction has reported the application of ultrasound to extract clove oil [9].

In this study, we concentrate on examining the effects of using microwave-assisted DESs and ultrasonic-assisted DESs for pretreatment extraction, in conjunction with microwave-assisted hydrodistillation (DES-MHD and U-DES-MHD), on the yield of essential oil extraction from clove buds, eugenol content, and environmental impact, in comparison to using microwave-assisted hydrodistillation (MHD) and ultrasound pretreatment before microwave-assisted hydrodistillation (U-MHD) without DES. Additionally, the produced clove essential oil’s antioxidant capacity was examined and contrasted with that of the other isolates.

## 2. Results and Discussion

### 2.1. Effect of Microwave Power and Time in the Pretreatment Stage of the DES-MHD Method

**Figure 1:**
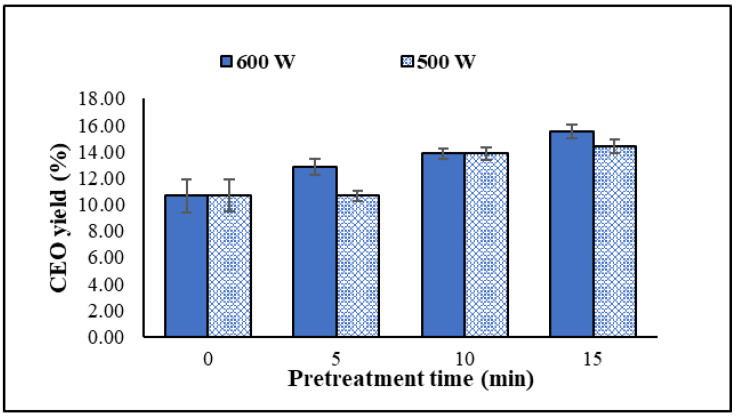
The effect of microwave power and time in the pretreatment state of the DES-MHD method (MHD condition [7]: no pretreatment state (0 min); distillation state, power 400 W, 45 min; pretreatment state (5–15 min), distillation state, power 400 W, 30 min).

According to the principle of green chemistry, this work was to find a more effective way to increase the CEO yield. Therefore, microwave-assisted DESs for pretreatment extraction, in conjunction with microwave-assisted hydrodistillation (DES-MHD), were examined in comparison to the MHD method. ChCl-EG (1:2) was used as a solvent in the pretreatment phase of the DES-MHD process to optimize microwave power and time for the highest oil output. Figure 1 showed that microwave pretreatment extraction before hydrodistillation increased the CEO yield. Pretreatment with microwave power (600 W) gave the oil a better yield than 500 W. High microwave energy can quickly raise the temperature, break the cellulose structure of clove cells, and aid in the solvent’s dissolution of more essential oil from the plant tissue [17]. The pretreatment state, using a microwave power of 600 W for 5–15 min, resulted in a slight increase in yield from 12.83% to 15.52%. This condition is slightly different from that reported by Chen et al. [8], where they used the same microwave power of 600 W in the pretreatment state of the DES-MHD method to extract clove oil. However, extending the pretreatment time beyond 5 min resulted in a slight decrease in the clove oil yield. Therefore, the ideal pretreatment conditions for the DES-MHD process involved using a microwave power of 600 W for 15 min, followed by adding water and distilling the oil using a microwave power of 400 W for 30 min. The maximum oil yield was obtained in 15.52% when ChCl-EG in 1:2 molar ratios was used as the solvent. The ratio of clove buds to DES (1:20) and the distillation stage of MHD were the same as that reported by our group before [7] when there was no DES.

### 2.2. Effect of DES Ratios on CEO Yields

The molar ratios of the DESs may influence the yield of the CEOs, depending upon the solubilizing capacity of each molar ratio of the DES for the clove bud cell wall. Consequently, the molar ratios of each type of DES (Table 1) were examined under similar conditions as the DES-MHD method. 

Table 1 lists all the prepared DESs and the relevant starting materials, including their molar ratios, the names of solvent abbreviations, as well as the pH values of DESs. DESs are formed by hydrogen bonding between the hydroxyl group of HBD and the ammonium chloride salt of choline chloride in specific molar ratios, resulting in a eutectic mixture that has a melting point lower than that of either component [24]. The pH values of each DES depended on the acidity of the HBD components. Ethylene glycol is less acidic than other compounds. The formation of DES at an appropriate ratio of ethylene glycol prevents some of its hydroxyl groups from releasing protons, resulting in a higher pH value than ethylene glycol alone. However, in the case of DESs (ChCl-G and Ch-F), the glycerol and fructose contain more hydroxyl groups than ethylene glycol, which contrib-utes to their high viscosity. Therefore, at higher molar ratios of HBD, their intermolecular hydrogen bonding interaction may cause ChCl-G (1:4) and Ch-F (1:2) to have a higher pH value than at lower molar ratios of these compounds.

**Figure 2:**
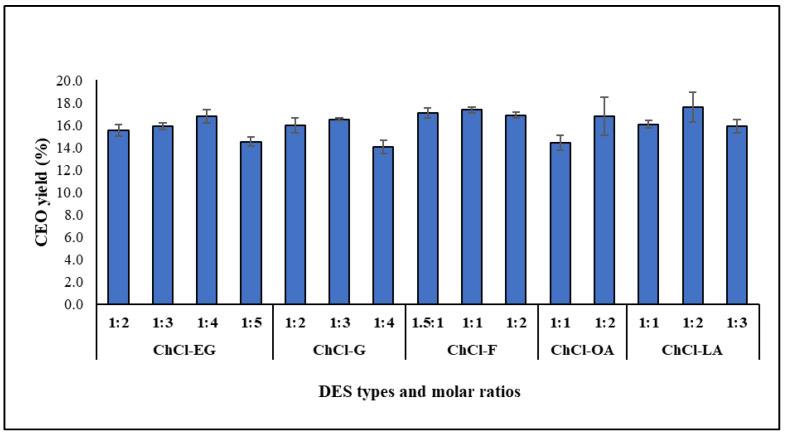
The effect of DES types and molar ratios on CEO yield by the DES-MHD method (condition: pretreatment state, power 600 W, 15 min; distillation state, power 400 W, 30 min).

Figure 2 illustrated that the optimal molar ratios of ChCl-EG, ChCl-G, ChCl-F, ChCl-OA, and ChCl-LA for extracting CEO were 1:4, 1:3, 1:1, 1:2, and 1:2, respectively. This gave a CEO yield of 16.49 to 17.60%. The differences in the molar ratios of each DES type for extraction efficiency are attributed to the physical properties of each HBD. These properties include polarity, viscosity, acidity, and hydrogen bond interactions that can occur between HBAs or natural compounds [15,16,25,26,27,28]. The extraction process using the DES type of ChCl-LA produced the highest CEO yield. The strong acidity and low viscosity of lactic acid resulted in the formation of DES from chlorine chloride and lactic acid as an acid solution, thereby enhancing the extraction efficiency of CEOs. The acid solvent could break down the cell wall faster and let out natural components [29]. If it were stronger, it might have been able to decompose sensitive compounds in essential oils like terpenes and esters when heated, which would have resulted in a lower yield. Because of this, the DES type of ChCl-OA had a lower CEO extraction yield than the DES type of ChCl-LA. The acidity of all DES solutions is shown in Table 1, and it was found that the ChCl-OA solution is more acidic than the ChCl-LA solution.

The selected molar ratios of five DESs, ChCl-EG (1:4), ChCl-G (1:3), ChCl-F (1:1), ChCl-OA (1:2), and ChCl-LA (1:2), were employed as solvents for the extraction of CEOs via the U-DES-MHD method. Recent research has utilized ChCl-LA (1:2), ChCl-EG (1:2), and ChCl-F (1:2) as pretreatment solvents in conjunction with the MHD technique to extract essential oil from clove buds. ChCl-LA (1:2) provided the highest essential oil yield of 4.6% [8]. Furthermore, ChCl-LA (1:2), ChCl-OA (1:1), and ChCl-F (3:2) were used as pretreatment solvents coupled with the MHD method for extracting essential oil from turmeric; the resulting ChCl-OA (1:1) gave the maximum essential oil yield [21]. The DESs ChCl-EG (1:4) and ChCl-G (1:2) had been employed as pretreatment solvents combined with the MHD method for the extraction of essential oil from Amomum fruits, resulting in ChCl-EG (1:4) being a better solvent than ChCl-G (1:2) [18]. However, the DES form ChCl-G (1:3) was utilized as an extraction solvent in the ultrasound-assisted extraction of polyphenols from agri-food waste biomass [30]. Additionally, ChCl-G (1:2) has been employed as an extraction solvent in the ultrasound-assisted extraction of phenolic compounds from clove [9]. DES form ChCl-OA (1:2) has been employed as an extraction solvent for the extraction of flavonoids from Aurantii Fructus (AF), the fruit of *Citrus aurantium* L. [31]. The DES form ChCl-F (1:1) has been used as a storage medium for a therapeutic protein [32].

### 2.3. Comparison of CEO Yields Obtained by DES-MHD and U-DES-MHD

**Figure 3:**
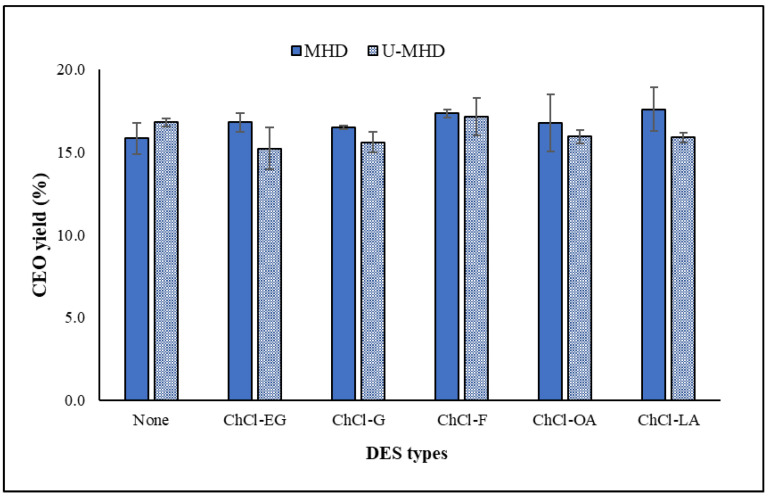
Effect of DES types for MHD and U-MHD methods on CEO yields (MHD condition: pretreatment state, power 600 W, 15 min; distillation state, power 400 W, 30 min. U-MHD condition [7]: pretreatment state, ultrasonic temperature 40 °C, 45 min; distillation state, microwave power 400 W, 30 min).

The impact of employing ultrasonic-assisted DESs for pretreatment extraction, coupled with microwave-assisted hydrodistillation (U-DES-MHD), was analyzed relative to the DES-based MHD method. According to Table 2 and Figure 3, the extraction yields of CEOs using five DES compositions (ChCl-EG, ChCl-G, ChCl-F, ChCl-OA, and ChCl-LA) for pretreatment extraction with DES-MHD and U-DES-MHD methods ranged from 16.49 ± 0.11 to 17.60 ± 1.33% and 15.22 ± 1.27 to 17.15 ± 1.16%, respectively. While, the CEO yields extracted without DES by the MHD and U-MHD methods were 15.83 ± 0.95% and 16.80 ± 0.26%, respectively. All methods extracted essential oils with insignificant differences. The DES-MHD method achieved CEO yields that were slightly higher than the U-DES-MHD method and greater than the MHD method with no DES. However, with the U-DES-MHD method, only the DES type of ChCl-F proved slightly higher CEO yields than the oil extracted via U-MHD without DES. The findings align with the previous research by Zhang et al. [33], which demonstrated that the oil yield from the microwave-assisted DES pretreatment extraction pair with MHD was superior to that from the ultrasonic-assisted DES pretreatment extraction pair with MHD. Additionally, our findings align with the findings of Chen et al. [8], who reported that DES-based MHD yielded a higher extraction efficiency compared to using only water as the solvent in the MHD method. Furthermore, the CEO yields from our research using the DES-MHD method were higher than those previously reported by Chen et al. [8], who reported an oil yield of 4.6% from the DES-based MHD method.

The rise in oil yield through microwave-assisted DES extraction may be attributed to the effective solvation of DESs within the cellulose matrix of clove buds. Upon damage to the cellulose structure, more natural compounds with a hydrogen bond and van der Waals interactions with DES could be accelerated and released from the plant tissue into the solvent [16]. The extraction yield of CEOs is negatively impacted by ultrasonic-assisted DES pretreatment extraction. It is likely that the ultrasonic wave increases the release of compounds from plant tissue into the solvent [33]. Consequently, certain sensitive compositions of the essential oil in the solution may decompose under heating, leading to a lower yield.

The results of this study corroborate several previous reports highlighting the effectiveness of DESs in extracting essential oils. In the studies of Chen et al. [8], the DES group of choline chloride and lactic acid combined with MHD produced a higher yield of clove oil than the MHD method (4.6 vs. 2.5%). Using the identical extraction approach, Yu et al. [17] reported that the essential oil yields of white and black pepper fruit extracted by the DES group of choline chloride and fructose combined with MHD were slightly higher than the oil yields from the MHD method (1.78 vs. 1.72% for white pepper and 1.77 vs. 1.71% for black pepper). In the studies of Chen et al. [22], the ultrasonic-assisted DES group of choline chloride and malic acid followed by the hydrodistillation (HD) method produced higher yields of perilla oils than ultrasonic-assisted HD without DES (0.69 vs. 0.21%).

Compared to the conventional extraction of CEO by hydrodistillation (HD), as previously reported by our group [7], the DES-MHD and U-DES-MHD methods enhanced the extraction efficiency of the CEO yield, achieving a yield higher than the HD method by 2–5%, a quicker extraction period, and a reduced environmental impact. The use of ultrasonic-assisted DES pretreatment before the MHD method for separating clove oil has not been previously documented. Furthermore, this is the first time that ChCl-G and ChCl-OA have been reported as DES-based MHD methods for the separation of clove oil.

### 2.4. Composition of CEO Obtained by DES-MHD and U-DES-MHD

**Figure 4:**
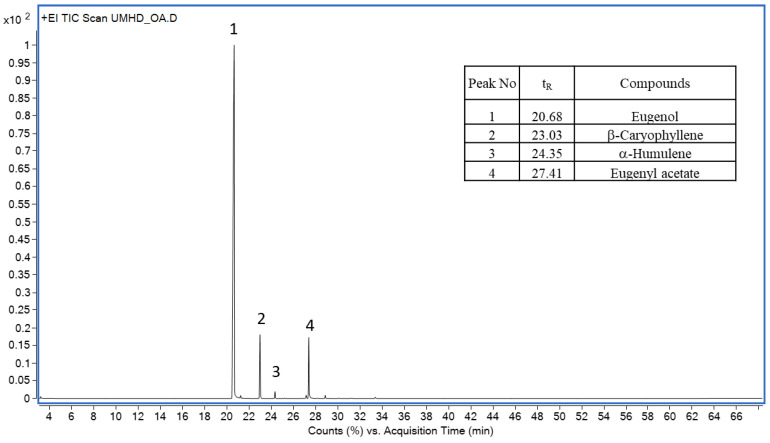
GC chromatogram of CEO from the U-DES-MHD method.

**Figure 5:**
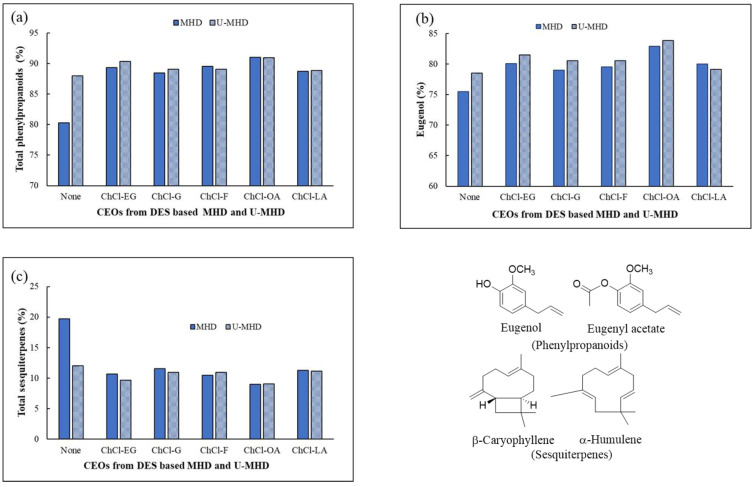
CEOs’ chemical compositions were extracted using the DES-MHD and U-DES-MHD methods. (**a**) Total phenylpropanoid compositions. (**b**) Eugenol compositions. (**c**) Total sesquiterpenes.

The constituents of twelve CEOs from DES-MHD, U-DES-MHD, and the untreated DES of both approaches were identified using GC/MS. A total of four major components, eugenol, β-caryophyllene, α-humulene, and eugenyl acetate, were found in the CEOs obtained via different extracts, as shown in the GC chromatogram (Figure 4). Anyway, the CEOs from different methods had the same major compounds, but their composition percentages varied. Table 2 summarizes the results, while Figure 5 illustrates the comparison. The CEOs from the DES-MHD process exhibited higher levels of eugenol components (78.97–82.90%) and total phenylpropanoids (88.44–91.02%) compared to those from the MHD method without DES pretreatment (75.48 and 80.30%). Also, the CEOs from the U-DES-MHD method had higher amounts of eugenol (79.14–83.84%) and total phenylpropanoids (88.89–90.97%) than the CEOs from the U-MHD method with untreated DES (78.49 and 88.01%). Both methods showed lower amounts of sesquiterpenes, β-caryophyllene, and α-humulene (8.98–11.56%) compared to CEOs that were extracted without DES. The DES-MHD and U-DES-MHD techniques produced CEOs with slightly different amounts of eugenol and total phenylpropanoids. However, when compared to CEO from classical HD, which had a eugenol composition of 72.48% and a total phenylpropanoids composition of 78.56%, as previously reported [7], all CEOs from both methods contained greater amounts of eugenol and total phenylpropanoids.

In both methods, the CEOs that were extracted using the DES type of ChCl-OA had the most phenylpropanoids with the highest eugenol composition and the least amount of sesquiterpenes. The eugenol content of the CEOs extracted using ultrasonic-assisted ChCl-OA-based MHD was slightly higher than that of the CEOs extracted using the ChCl-OA-based MHD (83.84 and 82.90%, respectively). Interestingly, the eugenol content of their oils was higher than that of the CEOs with untreated DES from the HD and MHD methods, with levels ranging from 10 to 11% and 7 to 8%, respectively.

The finding indicated that, in comparison to oils that were not treated with DES, DESs enhanced the ratio of phenylpropanoids to sesquiterpenes. Additionally, the U-DES-MHD method enhanced the eugenol composition compared to the oils with the DES-MHD method. It is probable that the DESs stimulate the release of oxygenated components, like phenylpropanoids, from the clove bud into the solvent due to their increased solubility through hydrogen bonding than nonoxygenated components, like sesquiterpenes [16]. However, the CEOs obtained by DES, which are composed of ChCl-OA, had the lowest amounts of eugenyl acetate. The reason likely stemmed from the strong acidity of oxalic acid, which served as an effective catalyst in the ChCl-OA water solution for the hydrolysis of ester, including eugenyl acetate. This leads to a decrease in the composition of eugenyl acetate, but it also contributes to an increase in the composition of eugenol. Similarly, the utilization of ultrasonic-assisted DES pretreatment extraction in the U-DES-MHD method also raises the amount of eugenol, because the ultrasonic wave accelerates the hydrolysis reaction of eugenyl acetate, which makes the eugenol content higher and the eugenyl acetate content lower [33]. Eugenol provides a vast diversity of uses due to its various biological characteristics. Reports indicate that eugenol, at low doses, can effectively treat several diseases without causing damage [4,5].

Our findings are consistent with previous studies that reported an increase in phenolic composition when DESs were used to extract natural compounds [28,34,35,36]. According to the research of Jeong et al. [34], the total phenolics and flavonoids in peppermint leaf extract obtained by DESs were higher than those of the extract obtained by water and alcohols. Similarly, Wojeicchowski et al. [35] found that the total phenolics in the rosemary leaf extract obtained by DESs were higher than those of the extract obtained by 70% aqueous alcohol. A study by Ojeda et al. [36] found that mango peel and seed extracts made with DESs had higher amounts of total phenolics and total flavonoids than extracts made with methanol and ethanol. Another study by Ratanasongtham et al. [28] discovered that the Thai colored rice bran extracts obtained by DES had more total anthocyanins, phenolics, and flavonoids than the extract obtained by 60% aqueous methanol.

### 2.5. DPPH Radical Scavenging Activity of CEOs

**Figure 6:**
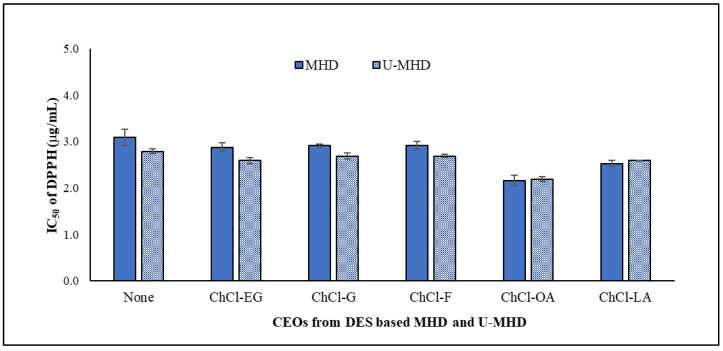
The antioxidant activities of CEOs were extracted using the DES-MHD and U-DES-MHD methods.

Twelve CEOs obtained from DES-MHD, U-DES-MHD, and the untreated DES of both methods were investigated for their DPPH radical scavenging activity. The results are presented in Table 2, and the comparison is demonstrated in Figure 6. The CEOs from the DES-MHD and U-DES-MHD processes had strong antioxidant activity, as shown by their IC_50_ values, which were 2.16 ± 0.11 to 2.92 ± 0.09 μg/mL and 2.19 ± 0.05 to 2.69 ± 0.06 μg/mL, respectively. These values were similar to those of eugenol (IC_50_ level of 2.35 ± 0.08 μg/mL) and Trolox (IC_50_ level of 2.89 ± 0.07 μg/mL). Their activities had a higher level than the oils with untreated DES, obtained by MHD (IC_50_ value of 3.09 ± 0.17 μg/mL) and U-MHD (IC_50_ level of 2.79 ± 0.05 μg/mL), respectively.

The CEOs extracted with the DES type of ChCl-OA from both methods showed the greatest activity, which corresponded to the highest amount of eugenol. These oils demonstrated significant antioxidant activity, comparable to pure eugenol, and significantly outperformed the CEOs with untreated DES from the MHD and U-MHD methods. Also, their oils were significantly stronger than the CEO extracted using traditional HD, which had an IC_50_ value of 3.38 ± 0.08 μg/mL for DPPH radical scavenging activity, as was already reported [7]. According to a comparison of the antioxidant properties in our research with clove essential oil extracted by the MHD process reported by Leon-Méndez et al. [37], we found that the CEOs obtained by DES extraction from our group had higher amounts of eugenol (79.52–83.34%) and greater DPPH inhibition (IC_50_ of 2.16–2.92 μg/mL) than CEOs extracted by the MHD process (eugenol of 65.0% and IC_50_ of 167.3 μg/mL), as reported by Leon-Méndez et al. [37].

According to the literature [28,34,35,36], DESs are widely used for extracting antioxidant compounds from various plants, including Thai colored rice bran [28], peppermint leaf [34], rosemary leaf [35], and mango peel and seed [36]. They had the ability to enhance antioxidant activity due to their efficiency in increasing phenolic compounds compared to conventional solvents, like alcohol and water. However, only one publication has reported on the antioxidant properties of clove oil extracted from DES [9]. They reported that the ultrasonic-assisted extraction of clove oil using choline chloride (ChCl)–propylene glycol-based DESs was more effective as an antioxidant than ChCl-based urea and glycerol, respectively. The findings showed that the CEOs extracted using DES, a non-toxic solvent, can serve as natural antioxidants and sources of eugenol for applications in food, cosmetics, and pharmaceuticals.

### 2.6. Environmental Impact

**Figure 7:**
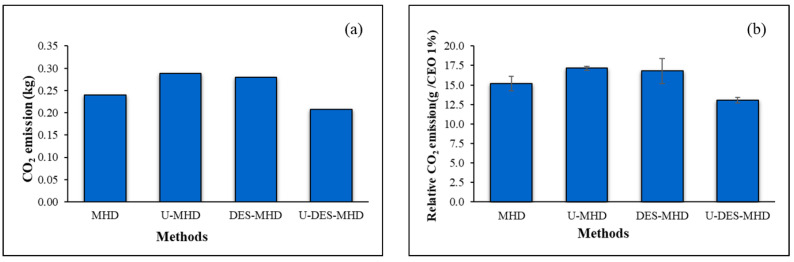
The environmental impact of CEO extractions varies depending on the method. (**a**) CO_2_ emission (kg) and (**b**) relative CO_2_ emission (g/CEO 1%).

Figure 7 illustrates the environmental impact of CEO extractions using various methods by comparing them to CO_2_ emissions. For this study, the electrical consumptions required for the MHD, U-MHD, DES-MHD, and U-DES-MHD methods were 0.30, 0.36, 0.35, and 0.26 kW h, respectively. This resulted in CO_2_ emissions of 0.24, 0.29, 0.28, and 0.21 kg, respectively. Since the yield of CEOs obtained by different methods was not the same, the relative CO_2_ emissions for a yield of 1% were described in order to compare the environmental impact. The CEO yields from the DES type of ChCl-OA with MHD and U-MHD methods, which had the highest amount of eugenol and greatest activity, were analyzed to compare with untreated DES. The CO_2_ emissions for the MHD, U-MHD, DES-MHD, and U-DES-MHD methods were 15.2 ± 0.9, 17.1 ± 0.3, 16.8 ± 1.6, and 13.1 ± 0.3 g per yield of 1% CEO, respectively. Based on CO_2_ emissions, the U-DES-MHD method reduced CO_2_ emissions by 1.4 times when compared with U-MHD, while the DES-MHD showed slightly less CO_2_ emissions compared to the U-MHD. Likewise, the relative CO_2_ emissions for the U-DES-MHD method decreased by 1.3 times when compared with the U-MHD method, and the DES-MHD was slightly lower than the U-MHD method.

According to [7], the traditional HD method released 1.68 kg of CO_2_ and 131.0 ± 14.3 g per 1% CEO for relative CO_2_ emissions. On the other hand, the DES-MHD and U-DES-MHD methods used less energy and produced CO_2_ emissions that decreased by 6 and 8 times, respectively. Similarly, the DES-MHD and U-DES-MHD approaches reduced the relative CO_2_ emissions by 8 and 10 times, respectively, compared to the HD method.

Therefore, utilizing the U-DES-MHD technique to extract CEO could result in energy savings and a decrease in environmental impact due to lower CO_2_ emissions compared to U-MHD and classical HD methods, as previously described by our group [7]. Moreover, the U-DES-MHD technique produced CEO with a higher eugenol composition, the main bioactive compound, compared to other hydrodistillation methods using untreated DES [7]. Since DESs are considered green solvents, the CEO extracted from them is safe for direct human consumption and beneficial for the food and pharmaceutical industries.

## 3. Materials and Methods

### 3.1. Plant Parts and Chemicals

In this research, dried clove buds (*Syzygium aromaticum*) were obtained from a local market in Thailand and stored in a zippered plastic bag at room temperature. The material was ground into a fine powder and put in a sealed bag before the essential oil was extracted. The powdered clove bud was found to have an average moisture content of 13.06 ± 0.29%, as investigated using the approach outlined by Kusuma et al. [38]. Eugenol was obtained from the clove essential oil and quantified using spectroscopic methods. The compounds 2,2′-diphenyl-1-picrylhydrazyl (DPPH), 6-hydroxy-2,5,7,8-tetramethylchroman-2-carboxylic acid (Trolox), and an alkane standard solution (C_8_–C_20_) 40 mg/L in hexane were all taken from Sigma-Aldrich, St. Louis, MO, USA. The compounds 2,6-di-tert-butyl-4-methylphenol (BHT) was bought from Acros, Dreieich, Germany. Choline chloride (98%) was obtained from Loba Chemie Pvt. Ltd., Mumbai, Maharashtra, India. The ethylene glycol, glycerol, fructose, oxalic acid, and lactic acid were commercial grades that were purchased from a local company in Thailand.

### 3.2. Preparation of Deep Eutectic Solvents (DESs)

The DESs were prepared by mixing the hydrogen bond acceptor (HBA), choline chloride, with different kinds of hydrogen bond donors (HBDs), ethylene glycol, glycerol, fructose, oxalic acid, and lactic acids at a proper molar ratio that were modified by following the report of Yu et al. [17], as described in Table 1. The components were combined with vigorous stirring at 80 °C for 15–20 min, until a clear and homogeneous liquid appeared. The viscosity of DESs necessitated the addition of distilled water (50% *w*/*w*) before extracting clove essential oils. The pH of DESs was measured after water addition using a pH meter (Model FP20, Five Easy Plus, Mettler-Toledo, Greifensee, Switzerland).

### 3.3. Extraction of CEOs from Clove Buds

The microwave-assisted hydrodistillation (MHD) and ultrasound pretreatment prior to microwave-assisted hydrodistillation (U-MHD) procedures for CEO distillation were carried out in accordance with prior reports by our group [7]. The method of microwave-assisted DESs pretreatment coupled with MHD (DES-MHD) and ultrasonic-assisted DESs pretreatment prior to MHD (U-DES-MHD) consisted of two stages: the pretreatment stage and the hydrodistillation stage.

The DES-MHD process was as follows: In the pretreatment state, fine clove buds (10 g) were mixed with the prepared DESs (200 mL), as shown in Table 1, and put in a modified domestic microwave oven (ER-SGS34 TH, Toshiba, Bangkok, Thailand). The mixtures were pretreated by microwave irradiation at 500–600 W for 0–15 min (0 min means no pretreatment part). For the hydrodistillation state, 100 mL of distilled water was added into the flask and then heated using microwave irradiation under a power of 400 W for 30 min. The Clevenger-type apparatus received the distilled products, extracted them with dichloromethane, dried them over sodium sulfate anhydrous, and then, evaporated the dichloromethane under reduced pressure. The CEOs were obtained and stored in light brown vials at 4 °C until analysis. The CEO yield was defined as the ratio of the mass of oil to the mass of clove buds. The process extraction was carried out in triplicate. The microwave power and time during the pretreatment state, along with the molar ratios of the DES compositions, were all tuned for the highest possible oil output.

The U-DES-MHD extraction procedure, in the pretreatment state, five clove buds (10 g) were mixed with the optimal molar ratios of DESs (200 mL) from the DES-MHD method. The mixtures were ultrasonically extracted in an ultrasonic bath (Bandelin SONOREXTM (Berlin, Germany), frequency 35 kHz) at a temperature of 40 °C for 45 min, following the U-MHD condition as an earlier report by our group [7]. For the hydrodistillation state, distilled water (100 mL) was added to the flask, then placed in the microwave oven, followed by hydrodistillation by microwave irradiation under a power of 400 W for 30 min. Subsequently, the CEOs were then extracted following the same procedure described for DES-MHD.

### 3.4. GC/MS Analysis of CEOs

Each CEO solution in dichloromethane (20 mL/mL) was analyzed using an Agilent 7890B gas chromatograph combined with an Agilent 5977B mass spectrometer (GC/MS), operating with EI ionization at 70 eV. A 1 mL sample of the CEO solution was subjected into a split/splitless inlet set at an injector temperature of 230 °C, with a split ratio of 1:100. Helium was the carrier, with a constant flow rate of 1 mL/min. Components in the oils were separated into a HP-5 MS capillary column using the same conditions as previously reported [7]. The relative quantities of compounds were determined in percentages based on the peak area in the total peak chromatogram. The constituents were identified by matching their mass spectra to those in the NIST 2017 library. Additionally, their identification was further confirmed by comparing their linear indices to a series of n-alkanes (C_8_–C_20_) under the same analytical settings.

### 3.5. DPPH Radical Scavenging Activity of CEOs

The DPPH radical scavenging activities of the CEOs were examined based on the methods reported by our study’s document [7]. A 100 μM DPPH methanolic solution (1 mL) was combined with the CEO solutions in methanol (1 mL) that had been diluted by 5 and 50 ppm. The mixtures were incubated in a dark cabinet for 30 min before measuring them at 517 nm against DPPH control. The concentration of each CEO was calculated as a percentage of DPPH radical inhibition, following the Equation (1).

DPPH Inhibition (%) = (1 − A_s_/A_c_) × 100 (1)

where A_s_ is the absorbance of the CEO sample, and A_c_ is the absorbance of the DPPH control. For comparison, eugenol, Trolox, and butylated hydroxytoluene (BHT) were also assessed. The activities of the analyzed CEO were expressed as an IC_50_ level, indicating the concentration required to inhibit the DPPH radical by 50%, determined as defined in Diloksumpun et al. [39]. All analyses were carried out in triplicate, and the data were presented as a mean ± SD.

### 3.6. Environmental Impact

The environmental impact of MHD, U-MHD, DES-MHD, and U-DES-MHD processes for CEO extractions was determined concerning electrical consumption and CO_2_ emissions. Electrical consumption (A) and CO_2_ emissions (E_CO2_) were calculated using Equations (2) and (3), as described in prior research [7].

A = P × t(2)

E_CO2_ = (A × 800)/1000 (3)

where A is electrical consumption (kWh), E_CO2_ is CO_2_ emission (kg), P is electrical power (kW), and t is time (h).

Furthermore, the relative electrical consumption refers to the electrical consumption per 1% of the CEO obtained. The relative CO_2_ emission refers to the CO_2_ emission per 1% of the CEO gained.

### 3.7. Statistical Analysis

The CEO yields and DPPH radical scavenging activities were conducted in triplicate, with results expressed as means ± SD. The ANOVA procedure analyzed variation, while Tukey’s pairwise comparison test identified statistically significant differences (*p* < 0.05). The analysis utilized the trial version of Minitab 18.

## 4. Conclusions

This research successfully enhanced the eugenol composition in CEOs by combining DESs with ultrasound and microwave techniques, thereby improving the hydrodistillation of essential oil from clove buds. Microwave-assisted DESs for pretreatment extraction combined with microwave-assisted hydrodistillation (DES-MHD) effectively increased the yields of CEOs, improved the eugenol compositions, and corresponded to stronger antioxidant activity when compared to MHD with only water as a solvent. However, the use of ultrasonic-assisted DESs for pretreatment extraction coupled with MHD (U-DES-MHD) has the potential to increase the eugenol composition in CEOs more than DES-MHD and MHD methods, while also causing a slight decrease in CEO yields. However, utilizing the U-DES-MHD method could save electrical energy and reduce the environmental impact compared to U-MHD. The microwave-assisted ChCl-LA (1:2)-based MHD, is the ideal pretreatment extraction for achieving the highest CEO yield. However, the ultrasonic- and microwave-assisted ChCl-OA (1:2) could increase the eugenol content in CEOs more than other kinds of DESs, and it corresponded to the strongest antioxidant activity, which was comparable to pure eugenol.

This research reports a novel process that compares microwave-assisted DES-based MHD (DES-MHD) and ultrasonic-assisted DES-based MHD (U-DES-MHD) for the extraction of CEOs. Furthermore, this study employs a DES mixture of choline chloride and oxalic acid as a pretreatment solvent, in conjunction with the MHD method, for the extraction of CEOs from clove buds. These innovative techniques offer a high-quality alternative extraction method that is efficient, enriched with eugenol, nontoxic, easy to operate, and eco-friendly, suitable for the future separation of other edible natural products for food and pharmaceutical applications.

## Figures and Tables

**Table 1:** The compositions and pHs of deep eutectic solvents (DESs) were used for extraction.

Solvent Abbreviation	DESs Composition		Molar Ratios	pH ^d^
	HBA ^a^	HBD ^b^	(mol/mol) ^c^	
ChCl-EG	Choline chloride	Ethylene glycol	1:2, 1:3, 1:4, 1:5	4.53, 4.67, 4.67, 4.57
ChCl-G	Choline chloride	Glycerol	1:2, 1:3, 1:4	4.54, 4.54, 4.76
ChCl-F	Choline chloride	Fructose	1.5:1, 1:1, 1:2	2.90, 3.02, 3.75
ChCl-OA	Choline chloride	Oxalic acid	1:1, 1:2	0.33, 0.19
ChCl-LA	Choline chloride	Lactic acid	1:1, 1:2, 1:3	1.56, 1.25, 1.16

^a^ HBA (hydrogen bond acceptor); ^b^ HBD (hydrogen bond doner); ^c^ molar ratios were modified by following the report of Yu et al. [17]; ^d^ pH of DESs after adding water (50% *w*/*w*).

**Table 2:** The yields, compositions, and antioxidant activities of CEOs extracted by DES-MHD and U-DES-MHD compared with no DES.

DESs	Methods	Yield ^a^ (%*w*/*w*)	Composition (%) ^b^						IC_50_ ^c^ (μg/mL)
			E	C	H	EA	TP	TS	
None (water)	MHD	15.83 ± 0.95 a	75.48	17.15	1.78	4.82	80.30	19.70	3.09 ± 0.17 a
	U-MHD	16.80 ± 0.26 a	78.49	10.86	1.13	9.52	88.01	11.99	2.79 ± 0.05 bc
ChCl-EG	MHD	16.80 ± 0.59 a	80.09	9.70	0.97	9.24	89.33	10.67	2.88 ± 0.09 ab
	U-MHD	15.22 ± 1.27 a	81.46	8.82	0.86	8.86	90.32	9.68	2.59 ± 0.07 cd
ChCl-G	MHD	16.49 ± 0.11 a	78.97	10.46	1.10	9.47	88.44	11.56	2.91 ± 0.03 ab
	U-MHD	15.61 ± 0.62 a	80.55	9.99	0.98	8.48	89.03	10.97	2.69 ± 0.06 b–d
ChCl-F	MHD	17.34 ± 0.25 a	79.52	9.53	0.93	10.01	89.54	10.46	2.92 ± 0.09 ab
	U-MHD	17.15 ± 1.16 a	80.53	9.76	0.98	8.74	89.03	10.97	2.69 ± 0.03 b–d
ChCl-OA	MHD	16.78 ± 1.71 a	82.90	8.11	0.87	8.12	91.02	8.98	2.16 ± 0.11 f
	U-MHD	15.95 ± 0.40 a	83.84	8.17	0.86	7.63	90.97	9.03	2.19 ± 0.05 f
ChCl-LA	MHD	17.60 ± 1.33 a	79.99	10.20	1.06	8.74	88.73	11.27	2.53 ± 0.06 de
	U-MHD	15.89 ± 0.30 a	79.14	10.06	1.05	9.76	88.89	11.11	2.59 ± 0.01 cd

^a^ % Weight of CEO per weight of clove bud, data are represented as mean ± SD in triplicate. ^b^ % Composition was relative amount in all identified compounds by GC/MS, calculated from peak area. E (Eugenol, peak 1), C (β-Caryophyllene, peak 2), H (α-Humulene, peak 3), EA (Eugenyl acetate, peak 4), TP (Total phenylpropanoids), TS (Total sesquiterpene). ^c^ IC_50_, DPPH radical scavenging activity. Data are represented as the mean ± SD in triplicate. IC_50_ of positive control (eugenol was 2.35 ± 0.08 ef, Trolox was 2.89 ± 0.07 ab, BHT was 10.92 ± 0.50). Lowercase letters in the same column as % yield and IC_50_ represent significant differences among methods according to Tukey’s pairwise comparison test (*p* < 0.05).

## Data Availability

Data are contained within the article.
